# Sequence Comparison Alignment-Free Approach Based on Suffix Tree and *L-Words* Frequency

**DOI:** 10.1100/2012/450124

**Published:** 2012-09-10

**Authors:** Inês Soares, Ana Goios, António Amorim

**Affiliations:** ^1^Faculdade de Ciências da Universidade do Porto, 4169 Porto, Portugal; ^2^Instituto de Patologia e Imunologia Molecular da Universidade do Porto, 4200 Porto, Portugal; ^3^Centro de Matemática da Universidade do Porto, 4169 Porto, Portugal

## Abstract

The vast majority of methods available for sequence comparison rely on a first sequence alignment step, which requires a number of assumptions on evolutionary history and is sometimes very difficult or impossible to perform due to the abundance of gaps (insertions/deletions). In such cases, an alternative alignment-free method would prove valuable. Our method starts by a computation of a generalized suffix tree of all sequences, which is completed in linear time. Using this tree, the frequency of all possible words with a preset length *L*—*L-words*—in each sequence is rapidly calculated. Based on the *L-words* frequency profile of each sequence, a pairwise standard Euclidean distance is then computed producing a symmetric genetic distance matrix, which can be used to generate a neighbor joining dendrogram or a multidimensional scaling graph. We present an improvement to word counting alignment-free approaches for sequence comparison, by determining a single optimal word length and combining suffix tree structures to the word counting tasks. Our approach is, thus, a fast and simple application that proved to be efficient and powerful when applied to mitochondrial genomes. The algorithm was implemented in Python language and is freely available on the web.

## 1. Introduction

During the last decades many sequence comparison methods have been developed in order to recover evolutionary and phylogenetic signals as well as for the discovery of pathogenic mutations [1, 2].

The most common approaches are based on sequence alignments [3, 4]. However, alignment quality depends on the penalties attributed to observed differences between sequences during the alignment process [5, 6]. Alternatively, many alignment-free methods have also been proposed [5, 7–9] which, being based on word frequencies or on match lengths, are algorithmically simple and computationally faster than alignment methods.

The basis of word frequency tasks is the determination of the optimal word length, *L*, which should be computed a priori. The *L-words* counting in a sequence is usually performed considering a one base sliding window, overlapping *L* − 1 consecutive bases, that is, shifting one base each time until *m* − *L* + 1, *m* being the sequence length [7, 8].

Here, we present a new approach that determines a single optimal word length, *L*, and generates *L-words* frequency profiles using suffix tree theory. The algorithm was applied to a variety of mtDNA sequences that are particularly difficult to handle by automated alignment methods and the performance was compared to the available word counting alignment-free methodologies.

## 2. Methods

### 2.1. Algorithm

We present here a new algorithm representing an improvement of word counting alignment free methodologies. The algorithm is described in Supplementary Material available online at doi:10.1100/2012/450124 and each step is summarized below.

#### 2.1.1. Suffix Tree Approach

The first step of the method is the construction of a generalized suffix tree, *T*, of *n* sequences, *S*_1_, *S*_2_, …, *S*_*n*_, where every suffix in the data set is represented only once. Therefore, the memory requirements when using these structures are much more modest than when considering the original complete sequences. The construction of a generalized suffix tree is based on Ukkonen's algorithm, described with detail by Gusfield [10]. Function *GST* in the Supplementary Algorithm 1 automates the construction of this structure.

Generalized suffix trees are potent structures, having the useful property that each prefix of paths leading from the root to any internal node points to all occurrences of this prefix in the data set [10]. Thus, when aiming to determine the number of times that a word *w* occurs in each sequence, we only need to traverse the generalized suffix tree leading from the root in the direction of the branch labeled by a prefix of *w* − *w*[1,…, *j*], 1 ≤ *j* ≤ *L*. If such branch does not exist, we conclude that *w* does not occur in the data set. Otherwise, we must always skip from a node to its descendant until the end of *w*. The indexes of all descendant leafs from the last node reached, or from its descendant nodes, are used to determine the sequences in the data set which contain *w* as well as the number of occurrences of *w* in each sequence. Each leaf indexes the sequences and the corresponding starting positions of the associated suffixes labeled in the path that leads from the root to this leaf.

An alternative approach, using a k-truncated suffix tree deserves consideration, due to reduction in both memory requirements and running time [11].

#### 2.1.2. *L-Words* Frequencies

In the next step, we determine all words in the DNA alphabet {A, C, G, T} with length L—W_L_—determined a priori, following the method of Sims et al. [7]. According to these authors, there is an optimal resolution range in which any integer value should be considered as the length of *L*. Any value inside this interval is equally good. So, in order to increase the speed of the process we start by considering only the lower limit of resolution, which is given by the expression log_4_(*m*), where *m* is the sequence length. Considering *n* sequences with different lengths *m*, the expression log_4_(*m*) can obviously generate different values. In order to find a value applicable to all sequences under analysis, we choose *m* as the length of the greater sequence and *L* as the smaller integer greater than log_4_(*m*). Thus, in the present study, we work with the following values:

(1) m=max⁡{length(Si),1≤i≤n},

(2) $$\begin{array}{c} L=\left\lceil \text{lo}{\text{g}}_{4}\left(m\right)\right\rceil , \end{array}$$

where ⌈*x*⌉ is the *ceiling function of x*, defined as the smallest integer is not less than *x*.

Notice that the total number of possible *L-words* is *t* = 4^*L*^ and *W*_*L*_ = [*w*_*L*_1__, *w*_*L*_2__, *w*_*L*_3__,…, *w*_*L*_*t*__]. For example, if *L* = 2 then *t* = 16 and the following result is obtained:

(3) W2=[AA,AC,AG,AT,CA,CC,CG,CT,GA,GC,GG,GT,TA,TC,TG,TT].

Using the generalized suffix tree we can efficiently determine the number of occurrences of each *w*_*j*_ ∈ *W*_*L*_ in each sequence *S*_*i*_ just by traversing the branch with path label *w*_*j*_ from the root towards the leafs only one time, as was thoroughly explained in the previous section: *O*_*ij*_ = #{*w*_*j*_  in  *S*_*i*_}.

Finally, we can determine the relative frequency of each word *w*_*j*_ in each sequence *S*_*i*_ − *f*_*ij*_ as the following:

(4) $$\begin{array}{c} f_{ij}=\frac{O_{ij}}{\sum_{j=1}^{t}O_{ij}}\in \left[0,1\right]. \end{array}$$

The resulting matrix *F*_*L*_ with dimension *n* × *t* and entries *f*_*ij*_ represents a global profile of *L-words* frequencies of all input sequences. The determination of each element *f*_*ij*_ is automated with function *LwF* in the Supplementary Algorithm 1.

#### 2.1.3. Genetic Distance

The generated frequencies matrix may then be used to assign a pairwise correlation or a metric distance between each pair or sequences. In this work we calculate the pairwise standard Euclidean distance, which is defined as

(5) $$\begin{array}{c} SED\left(X,Y\right)=\sqrt{\sum_{w\in W_{L}}{\left(f_{X_{w}}-f_{Y_{w}}\right)}^{2}}\in \left[0,1\right], \end{array}$$

where *w* represents the *L-words* and *f*_*Z*_*w*__ means the relative frequency of *w* in the sequence *Z*.

Function *Distance* described in Supplementary Material automates this procedure.

### 2.2. Software

The algorithm was written in Python, version 2.5.2, and tested on a Windows 7 x32 system and on a Linux platform with a processor Intel (R) Pentium (R) Dual CPU, T3400 @ 2.16 GHz and 4 Gb of RAM. It is freely available on the web at http://www.portugene.com/SupMat/SuffixTree&Lwords.rar.

## 3. Results and Discussion

### 3.1. Phylogenetic Reconstructions

The developed algorithm was tested in different datasets of mtDNA sequences, proving to be a simple and fast way to identify phylogenetic relationships in the different sets of mitochondrial genomes.

The algorithm was first tested in a dataset composed of 29 complete primate mtDNA sequences representing genomes of different families, ranging from 15467 bp to 17036 bp long. Taking into account these lengths, we determined *L* = 8, as explained in the Methods Section. This value has proven to be a good choice, allowing the program to run quickly, while still producing a genetic distance matrix that, when used to construct a dendrogram, exhibits a clustering that is in agreement with consensus primate phylogeny (http://tolweb.org/Primates/15963). 

In order to confirm that the algorithm was also able to produce a correct phylogeny with closely related sequences we tested it with mtDNA sequences from the same species, in which the sequence length is more homogeneous. The observed clusterings are in general agreement with those published in the literature, grouping mtDNA genomes in the same clades previously published methodologies (Supplementary Material).

Aiming to check the performance of our algorithm as well as to compare the quality of the results obtained by our approach and Costa's methodology, we compare the topology of the resulting phylogenies. The dendrograms constructed using the genetic distance matrixes generated by our algorithm are consistent with consensus phylogenies (Supplementary Material), in contrast with the results obtained by Costa et al. [8] methodology, which show some discrepancies, namely, in the clade Platyrrhini, which is clustered with Tarsii and Strepsirrhini ((http://tolweb.org/Primates/15963) and [12]).

### 3.2. Running Time

Our algorithm takes a linear execution time to determine the words frequencies and a quadratic time to compute the pairwise distances, an improvement to previous word counting alignment-free methodologies.

Our approach was compared to the method developed by Costa et al. [8] in what concerns the running time (the word counting alignment-free methodology proposed by Sims et al. [7] could not be tested because it has not been made available). While our approach computes the optimal word length to determine the word frequency profiles and generates a genetic distance matrix just by inputting a *fasta* file with mtDNA sequences, the methodology proposed by Costa et al. [8] involves four steps/algorithms: (1) converting a *fasta* file containing *n* mtDNA sequences into *n fasta* files with a single sequence; (2) converting each file into a *fa* file, a simplified version of *fasta* files; running two additional algorithms to (3) generate the histograms files and (4) create a correlation similarity matrix. These last two algorithms must be tested in increasingly longer windows until a conserved correlation matrix is obtained.

**Figure 1:**
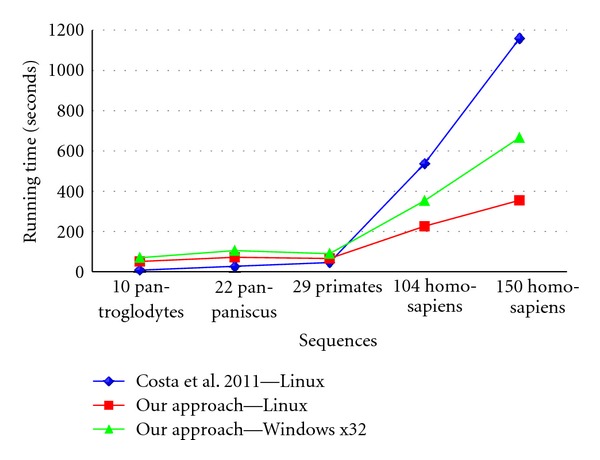
Differences between running times of Costa et al. 2011 [8] approach and our suggested methodology.

Our approach was designed to be run in Windows x32 operative system but it was also tested in a Linux platform in order to be compared to the alternative methodology under the same operative system. We thus could demonstrate that, independently of the operating system, the use of suffix tree structures to compute the words frequency profiles enables our methodology to run in a much shorter time. Although for small sets of sequences the running time required by Costa's (2011) methodology [8] is shorter, when increasing the number of sequences to over a hundred, the performance of our method is clearly better (Table 1, Figure 1, Supplementary Table 5).

### 3.3. Final Remarks 

The algorithm described here has demonstrated to be an improvement of word counting alignment-free methods for sequence clustering, showing to be computationally very fast, particularly with large datasets, while still producing good quality results. In fact, by combining suffix tree structures with word counting tasks, as well as automating the determination of a single optimal word length, a significant decrease in running time and memory requirements for *L-words* frequencies determination was obtained. 

The method proved to be efficient and powerful when applied to complete mitochondrial genomes, either from different species or intraspecifically, being able to quickly cluster the sequences in accordance to acknowledged phylogenetic relationships.

## Supplementary Material

Complementary text.

## Figures and Tables

**Table 1:** Comparison of the running times between our approach (Linux and Windows x32 operative systems) and Costa et al. 2011 methodology (Linux platform) [8]. The first column lists the number of sequences and species used in each comparison; the second and third summarize the running times of each algorithm for each set of sequences, in seconds. The tabulated times correspond to the sum of running times of each step. The time spent by the user between each step, although highly time consuming, was not included.

	Running time (seconds)		
Sequences	Costa et al. 2011 [8]	our approach	
	Linux	Linux	Windows
10 Pan troglodytes	8	51	70
22 Pan paniscus	27	72	105
29 primates	46	66	90
104 Homosapiens	537	226	353
150 Homosapiens	1159	355	666
