# Supplementary material for: Sequence Comparison Alignment-Free Approach Based on Suffix Tree and L-Words Frequency
Source: ScientificWorldJournal. 2012 Sep 10;2012:450124. doi: 10.1100/2012/450124 (PMC3444837; doi:10.1100/2012/450124)
Supplement: Supplementary file 1 — Complementary text. [file 450124.f1.pdf]

SUPLEMENTARY MATERIAL

SYSTEM AND METHODS

Algorithm 1: Schematic illustration of the process to automate the estimation of pairwise distances by a word counting alignment-free approach.

Supplementary Algorithm 1 Estimation of pairwise distances by an alignment-free technique

Input: n mtDNA sequences, $S_1, S_2, \ldots, S_n$

Function **GST** ($S_1, S_2, \ldots, S_n$):

for $1 \le i \le n$:

    $m = length(S_i)$

    for $1 \le j \le m$:

        Actualize the tree T with the suffix $S_i[j \ldots m]$

end function

Function LwF (S_i):

$m = \max\{length(S_i) : 1 \le i \le n\}$

$L = \lceil \log_4 m \rceil$, where $\lceil x \rceil$ is the *ceiling function of* x, defined as the smallest integer not less than x

$t = 4^L$

$W_L = [w_{L1}, w_{L2}, w_{L3}, \ldots, w_{Lt}]$ where w_{Li} represents a word with length L with characters in $\{A, C, G, T\}$

for w_j in W_L:

traverse the branch with path label w_j from the root in generalized suffix tree to determine in which sequences w_j occurs and how many times – $O_{ij} = \#\{w_j \ in \ S_i\}$

for $1 \le i \le n$:

    for $1 \le j \le t$:

$$f_{ij} = \frac{O_{ij}}{\sum_{j=1}^{t} O_{ij}} \in [0,1]$$

end function

Function **Distance** (S_i, S_j):

for $1 \le i \le n$:

  for $1 \le j \le n$:

$$SED\left(S_i, S_j\right) = \sqrt{\sum_{a=1}^{t}\left(f_{S_i w_a} - f_{S_j w_a}\right)^2} \quad \in \quad [0,1]$$

end function

Output: Pairwise genetic distance matrix

Data Representation

Phylogenetic relationships were visualized through the representation of the data (genetic distance matrixes) in a dendrogram, through neighbor joining trees obtained generated using MEGA – version 4 (Tamura et al. 2007), or in a multidimensional scaling plot, using MDS – 2 D, generated on PERMAP – version 11.8 (Heady and Lucas 1997).

Sequences

The algorithm was tested in 4 training sets of complete mtDNA sequences: (i) 29 from different families of primates (Table 1) (ii) 22 from Pan paniscus (Table 2) (iii) 10 from Pan troglodytes (Table 3) and (iv) 104 from Homo sapiens, comprising representatives of all major haplogroups (Table 4).

Table 1: Identification of 29 mtDNA primate sequences used. Each line contains a numerical identification with the corresponding name of the organism and accession number.

Number of Sequence	Organism Name	Accession Number
1	Homo sapiens sapiens	NC_012920.1
2	Homo sapiens Neanderthalensis	NC_011137.1
3	Pan troglodytes	NC_001643.1
4	Pan paniscus	GU189661.1
5	Gorilla gorilla	NC_001645.1
6	Gorilla gorilla gorilla	NC_011120.1
7	Pongo pygmaeus	NC_001646.1
8	Pongo pygmaeus abelli	NC_002083.1
9	Hylobates lar	NC_002082.1
10	Hylobates agilis	NC_014042.1
11	Macaca mulatta	NC_005943.1
12	Macaca sylvanus	NC_002764.1
13	Papio hamadryas	NC_001992.1
14	Chlorocebus aethiops	NC_007009.1
15	Chlorocebus pygerythrus	NC_009747.1
16	Chlorocebus sabaeus	NC_008066.1
17	Chlorocebus tantalus	NC_009748.1
18	Colobus guereza	NC_006901.1
19	Pygathrix nemaeus	NC_008220.1
20	Pygathrix roxellana	NC_008218.1
21	Nasalis larvatus	NC_008216.1
22	Semnopithecus entellus	NC_008215.1
23	Presbytis melalophos	NC_008217.1
24	Trachypithecus obscurus	NC_006900.1
25	Cebus albifrons	NC_002763.1
26	Callicebus donacophilus	FJ785423.1
27	Tarsius bancanus	NC_002811.1
28	Lemur catta	NC_004025.1
29	Lepilemur hubbardorum	NC_014453.1

Table 2: Identification of 22 mtDNA *Pan paniscus* sequences used. Each line contains a numerical identification with the corresponding identification of the organism and the accession number. The identification of the organism is according to (Zsurka et al. 2010).

Number of Sequence	Organism Identification	Accession Number
1	PP03	GU189657
2	PP05	GU189658
3	PP06	GU189659
4	PP10	GU189660
5	PP23	GU189661
6	PP35	GU189662
7	PP54	GU189663
8	PP58	GU189664
9	PP56	GU189665
10	PP60	GU189666
11	PP68	GU189667
12	PP20	GU189668
13	PP14	GU189669
14	PP69	GU189670
15	PP61	GU189671
16	PP55	GU189672
17	PP57	GU189673
18	PP75	GU189674
19	PP11	GU189675
20	PP30	GU189676
21	PP18	GU189677
22	PP25	HM015213

Table 3: Identification of 10 mtDNA *Pan troglodytes* sequences used. Each line contains a numerical identification with the corresponding name and identification of the organism and the accession number. The organism identification is in agreement with (Stone et al. 2010).

Number of Sequence	Organism Name	Organism Identification	Accession Number
1	PanTroglodytesTroglodytes	Pt13	GU112738
2	PanTroglodytesVerus	Pt82	GU112739
3	PanTroglodytesSchweinfurthii	Pt96	GU112740
4	PanTroglodytesVersus	Pt105	GU112741
5	PanTroglodytesEllioti	Pt114	GU112742
6	PanTroglodytesVerus	Pt115	GU112743
7	PanTroglodytesVerus	Pt120	GU112744
8	PanTroglodytesSchweinfurthii	Pt161	GU112745
9	PanTroglodytesVerus	Jenny	X93335
10	PanTroglodytesVerus_reference	P.t.Reference	NC_001643

Table 4: Identification of 104 mtDNA *Homo sapiens* sequences used. Each line contains a numerical identification with the corresponding accession number and haplogroup classification (according to http://www.phylotree.org (van Oven and Kayser 2009); Soares et al. 2011).

Number of Sequence	Accession Number	Haplogroup
1	AF347008.1	L0
2	AF347009.1	L0
3	AY195777.1	L0
4	D38112.1	L0
5	AF346998.1	L0
6	AF346999.1	L0
7	AF346985.1	L0
8	AY195780.1	L0
9	AY195766.1	L2
10	AF346995.1	L2
11	AY195785.2	L2
12	AY195788.2	L2

Number of Sequence	Accession Number	Haplogroup
53	AY195786.2	A
54	AY195765.2	K
55	AY195768.2	W
56	AY195779.2	W
57	AY195745.2	T
58	AF346982.1	T
59	AY195767.2	T
60	X93334.1	U
61	AF346964.1	N*
62	AF347005.1	P
63	AF347002.1	P
64	AF347004.1	P

13	AF346976.1	L2
14	AF346977.1	L2
15	AY195776.2	L2
16	AF347000.1	L3
17	AY195764.1	U
18	AY195756.2	N
19	AF347003.1	Q
20	AF346965.1	M*
21	AF347014.1	L3
22	AY195782.2	L3
23	AY195784.2	L3
24	AF346967.1	L3
25	AF346980.1	L3
26	AF346994.1	L3
27	AF347015.1	L3
28	AY195755.2	G
29	AF346966.1	G
30	AY195762.2	G
31	AF346972.1	M
32	AF347010.1	D
33	AY195790.2	D
34	AF346989.1	D
35	AF346984.1	D
36	AF346990.1	D
37	AY195748.1	D
38	AY195761.1	Z
39	AF347012.1	C
40	AF347013.1	C
41	AY195753.2	C
42	AY195759.2	C
43	AF346979.1	C
44	AF346970.1	C
45	AY195763.2	C
46	AF346991.1	C
47	AY195772.2	C
48	AF346973.1	F
49	AY195769.2	I
50	AY195791.2	F
51	AY195760.2	A
52	AF346971.1	A
65	AY195773.1	X
66	AY195787.1	X
67	AY195778.2	J
68	AY195754.2	J
69	AF346983.1	J
70	AY195774.2	J
71	AF347001.1	B
72	AY195749.2	B
73	AF346963.1	N*
74	AF346988.1	U
75	AF346993.1	B
76	AF347007.1	B
77	AY195770.2	B
78	AF347011.1	B
79	AY195751.1	H
80	AY195792.2	Y
81	AY195758.2	H
82	AC_000021.1	H
83	J01415.2	H
84	AF347006.1	V
85	AF346978.1	HV0
86	AY195750.1	V
87	AY195781.2	V
88	AY195746.2	H
89	AY195752.2	H
90	AY195757.1	H
91	AF346974.1	H
92	AF346975.1	H
93	AY195747.2	H
94	AF346981.1	H
95	AY195775.2	H
96	AF346986.1	L1
97	AY195783.2	L1
98	AF346992.1	L1
99	AY195789.2	L1
100	AF346968.1	L1
101	AF346997.1	L1
102	AF346987.1	L1
103	AF346969.1	L1
104	AF346996.1	L1

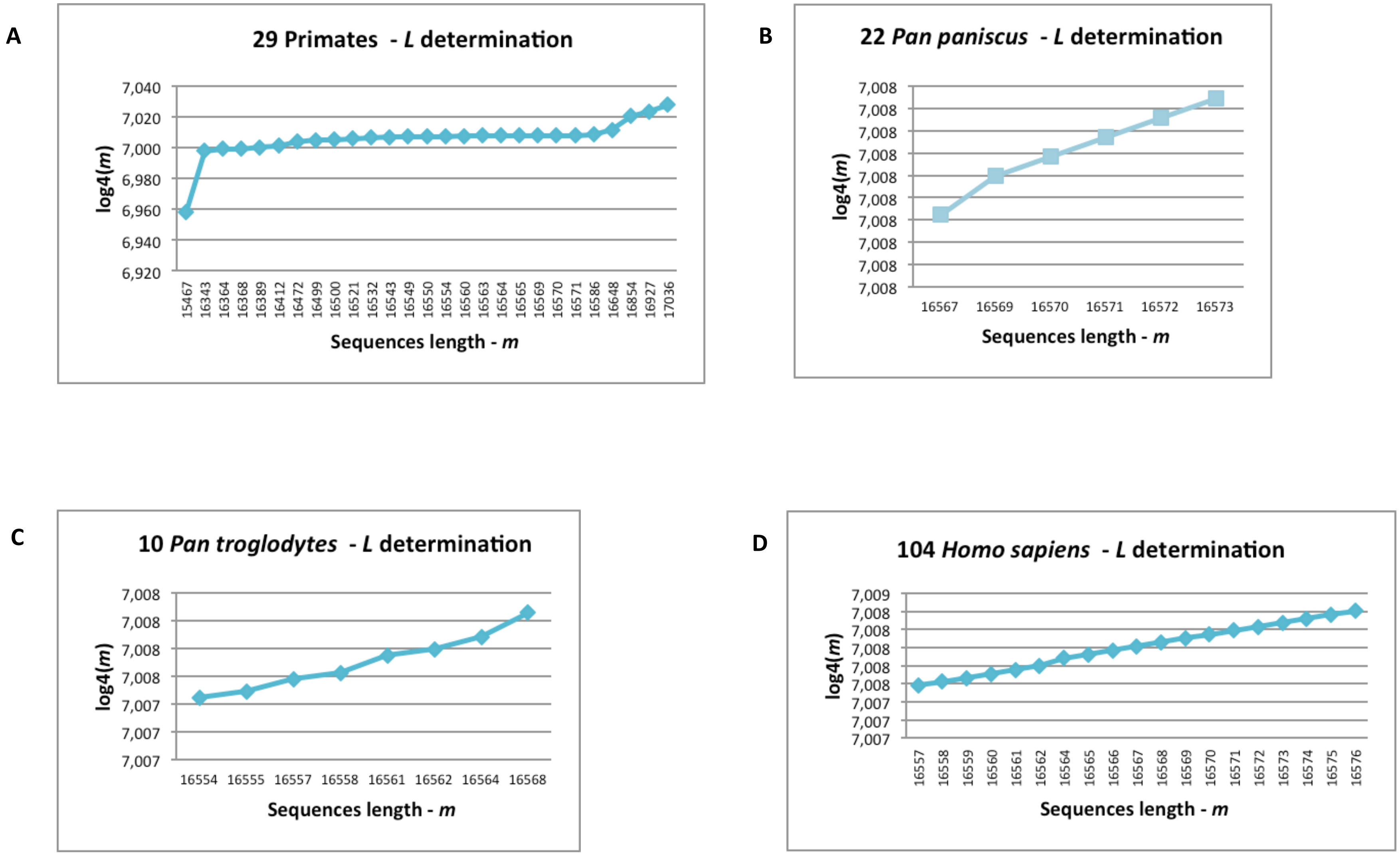

Figure 1: Words length determination used in the different data sets. (A) 29 Primatas. **(B)** 22 *Pan paniscus*. **(C)** 10 *Pan troglodytes*. **(D)** 104 *Homo sapiens*.

RESULTS

Phylogenetic reconstructions

The developed algorithm was tested in different data sets of mtDNA sequences.

The first test used a data set with 29 complete primate mtDNA sequences representing genomes of different families, ranging from 15467bp to 17036bp long. Taking into account these lengths, we determined $L=8$, as explained in the System and Methods section (Figure 1 A); this value allowed very fast runs, while still producing a genetic distance matrix in agreement with consensus primate phylogeny (Figure 2, http://tolweb.org/Primates/15963).

In order to confirm that the algorithm was also able to produce phylogenetically reliable results with closely related sequences we tested mtDNA sequences from the same species, in which the sequence length is much more homogeneous (Figure 1). We have analyzed three different data sets: (i) 22 complete Pan paniscus sequences (Figures 1 B and 3), (ii) 10 complete Pan troglodytes sequences (Figures 1 C and 4) and (iii) 104 complete Homo sapiens mtDNA sequences comprising representatives of all major human haplogroups (Figures 1 D and 5). The respective clusterings are in general agreement with those published in the literature (Tables 2 and 3) and with the human phylogeny as established in Phylotree (Table 4, http://www.phylotree.org). The observed clusterings are in general agreement with those published in the literature, grouping mtDNA genomes in the same clades as previously published methodologies (http://tolweb.org/Primates/15963; Zsurka et al. 2010, Stone et al. 2010; http://www.phylotree.org (van Oven and Kayser 2009); Soares et al. 2011).

Figure 2: Neighbor-Joining tree of 29 Primates mtDNA complete sequences comprising representatives of primate families. Genetic distance matrix was generated as described in *System and Methods* section, using *8-words*.

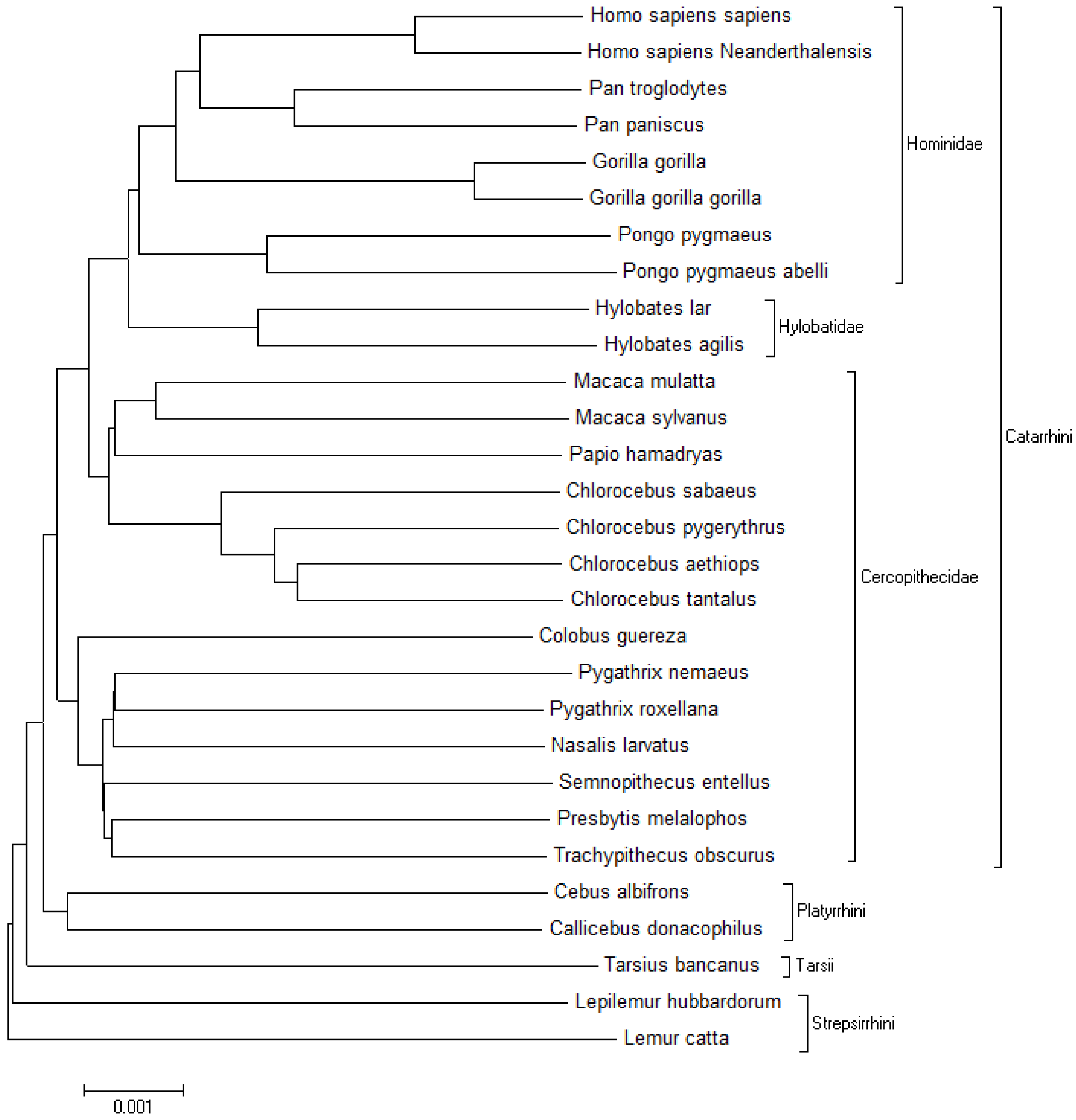

Figure 3: Neighbor-Joining tree of 22 *Pan paniscus* mtDNA complete sequences. Genetic distance matrix was generated as described in *System and Methods* section, using *8-words*. A, B and C are three diferent groups of bonobos. The nomenclature is in agreement with (Zsurka et al. 2010).

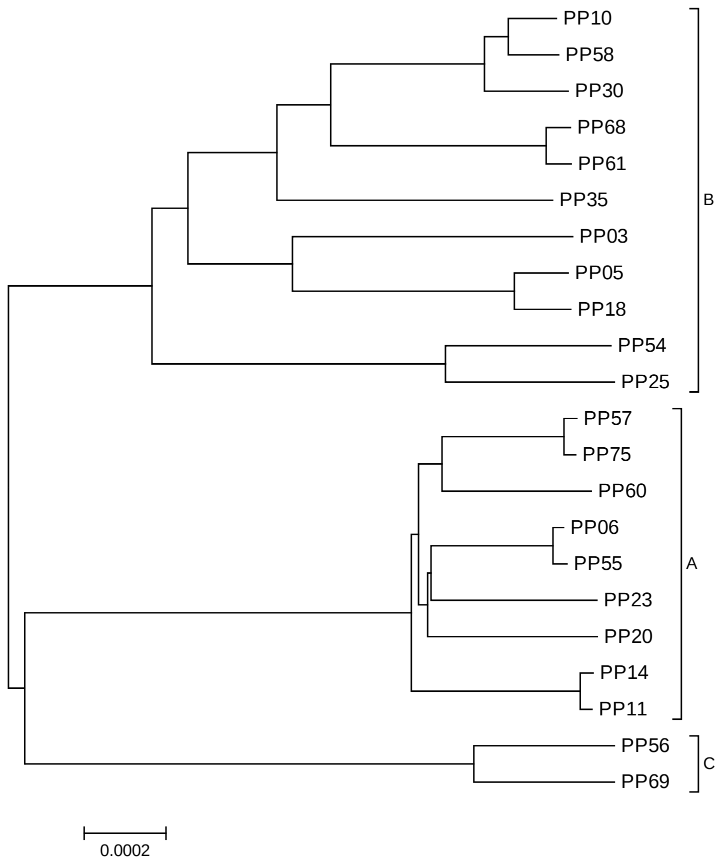

Figure 4: Neighbor-Joining tree of 10 *Pan troglodytes* mtDNA complete sequences. Genetic distance matrix was generated as described in *System and Methods* section, using *8-words*. The nomenclature is in agreement with (Stone et al. 2010).

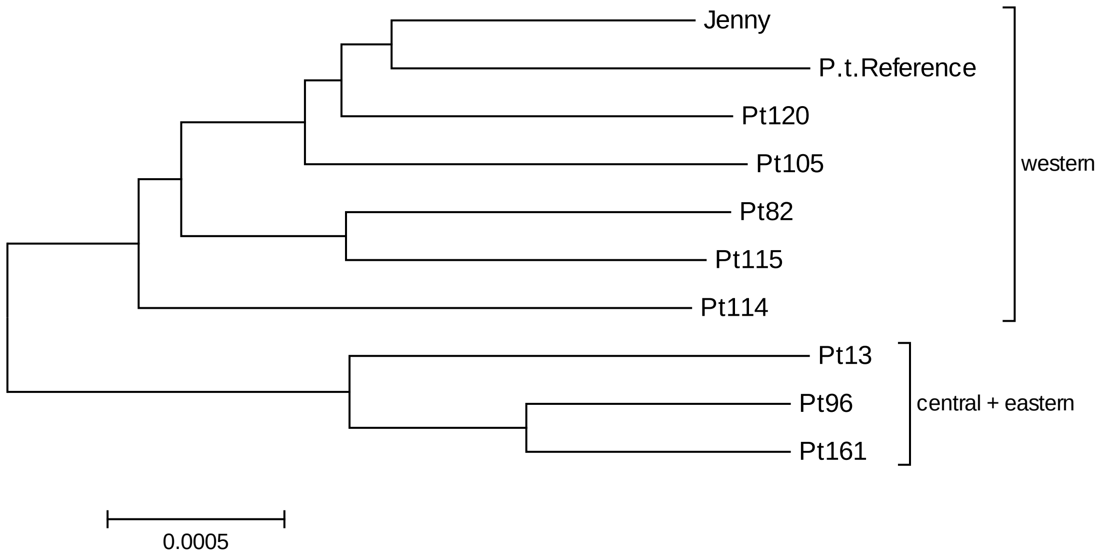

Figure 5: **Neighbor-Joining tree of 104 human mtDNA complete sequences comprising representatives of all major haplogroups.** Genetic distance matrix was generated as described in *System and Methods* section, using *8-words*. The labels contain a numerical identification for each sequence and the haplogroup classification (see Table 4).

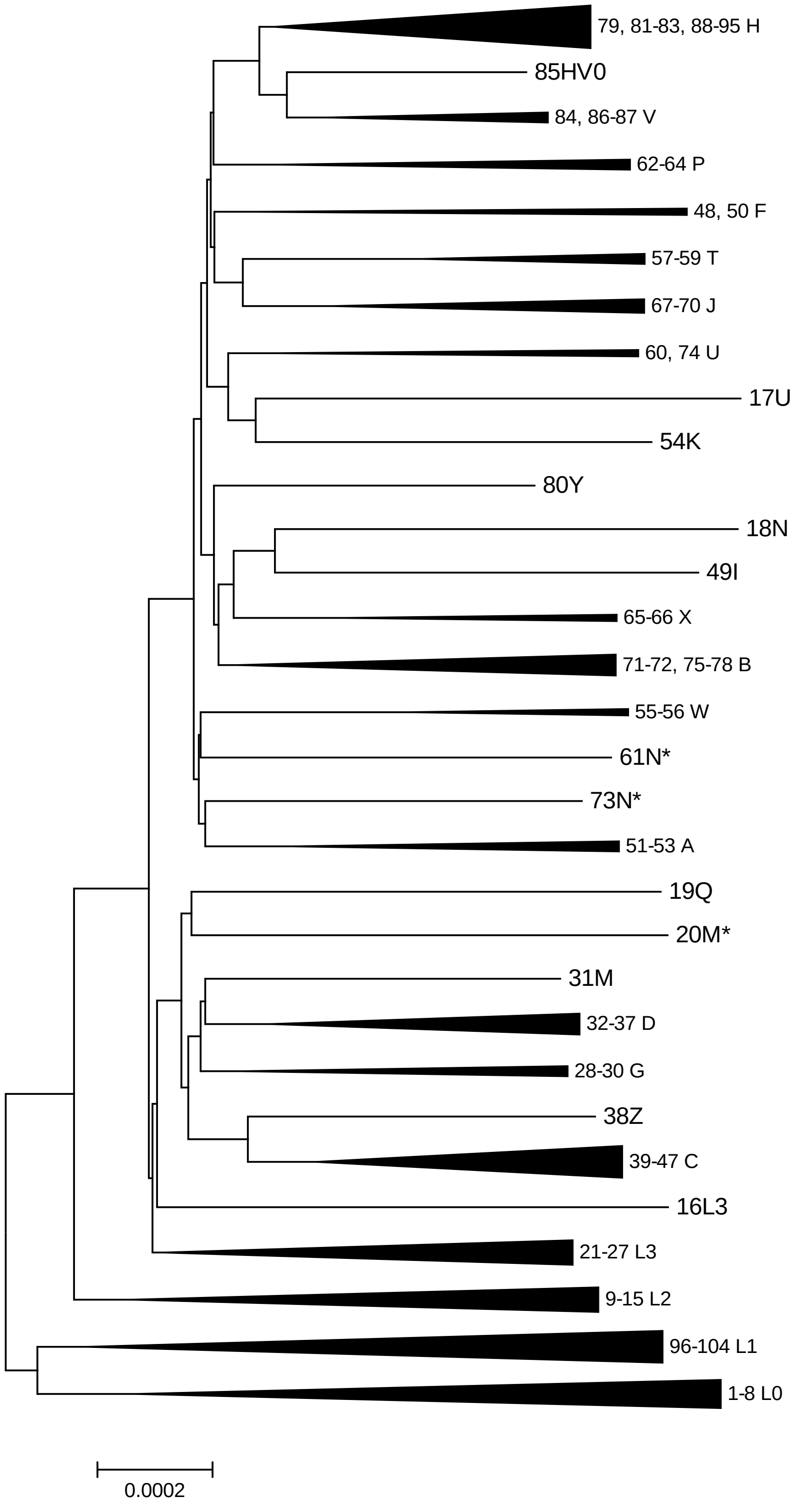

Figure 6: MDS-2D of 29 Primates mtDNA complete sequences comprising representatives of primate families. Genetic distance matrix was generated as described in *System and Methods* section, using *8-words*.

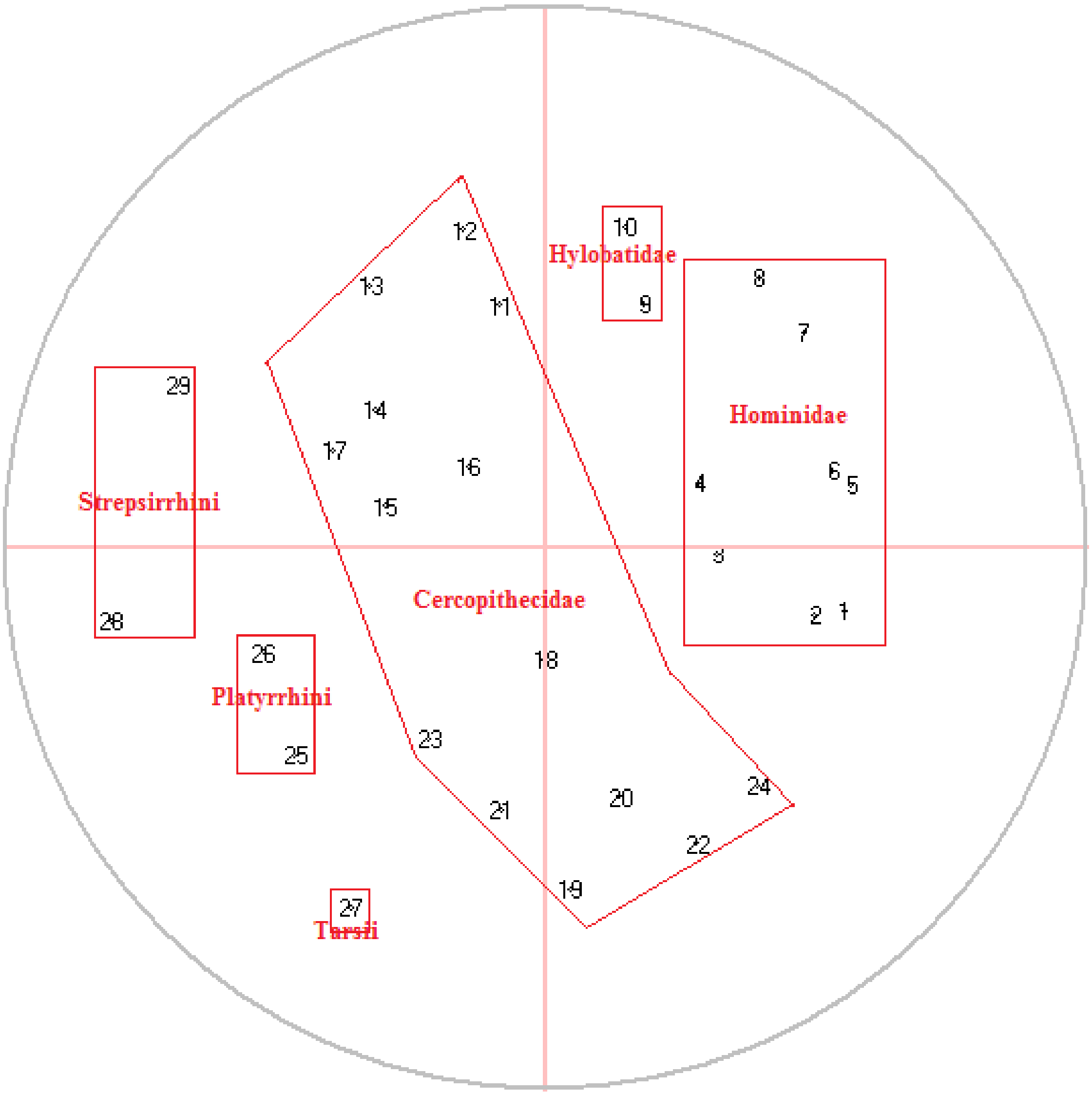

Running time

Table 5: Comparison of running times required by our approach to that from Costa et al. (2011). Costa's approach runs exclusively in Linux, while our proposed methodology works in both Linux and Windows systems. Costa's approach comprises 4 steps/algorithms: (i) conversion of each *fasta* file with *n* mtDNA sequences into *n* *fasta* files containing just one mtDNA sequence (the step is indicated as required by the authors but, since the corresponding algorithm is not delivered, an in-house developed one was used); (ii) conversion of each *fasta* file into a *fa* file; (iii) computation of the histogram files for each window length; (iv) computation of the correlation similarity matrix for each window length. The tabulated times correspond to the running times of each step being, at the end, summed all the summarized times. The time spent by the user between each step, although highly time consuming, was not included. The analyses of Costa's methodology ended when a window of length 8bp is considered. Our approach is performed in a single step, being the optimal window's length, 8bp, computed by our algorithm.

	Platform	Window	task/algorithm	Running Time				
				**10 Pan troglodytes**	**22 Pan paniscus**	**29 Primates**	**104 Homo spiens**	**150 Homo spiens**
Costa et al. 2011	LINUX	pre-processing work	*in-house created file*	1sec	1sec	1sec	2sec	2sec
			faclean.sh file	0.046sec+0.119sec = 0.165sec	0.094sec+0.249sec = 0.343sec	0.117sec+0.344sec = 0.461sec	0.409sec+1.077sec=1.486sec	0.604sec+1.744sec=2.348sec
			total	**1.165sec**	**1.343sec**	**1.463sec**	**3.486sec**	**4.348sec**
		1	*genhists file*	0.047sec	0.101sec	0.130sec	0.433sec	0.660sec
			gentauk file	0.179sec	0.934sec	1.589sec	19.454sec	40.638sec
			total	**0.226sec**	**1.035sec**	**1.719sec**	**19.887sec**	**41.298sec**
		2	*genhists file*	0.065sec	0.140sec	0.177sec	0.595sec	0.865sec
			gentauk file	0.185sec	0.883sec	1.636sec	19.422sec	41.096sec
			total	**0.250sec**	**1.023sec**	**1.813sec**	**20.017sec**	**41.961sec**
		3	*genhists file*	0.065sec	0.132sec	0.185sec	0.636sec	0.940sec
			gentauk file	0.180sec	0.875sec	1.648sec	20.063sec	41.165sec
			total	**0.245sec**	**1.007sec**	**1.833sec**	**20.699sec**	**42.105sec**
		4	*genhists file*	0.090sec	0.180sec	0.245sec	0.855sec	1.315sec
			gentauk file	0.200sec	0.948sec	1.791sec	21.572sec	44.419sec
			total	**0.290sec**	**1.128sec**	**2.036sec**	**22.427sec**	**45.734sec**
		5	*genhists file*	0.108sec	0.214sec	0.280sec	0.989sec	1.538sec
			gentauk file	0.241sec	1.089sec	2.000sec	24.777sec	51.162sec
			total	**0.349sec**	**1.304sec**	**2.280sec**	**25.766sec**	**52.700sec**
		6	*genhists file*	0.153sec	0.331sec	0.430sec	1.638sec	2.383sec
			gentauk file	0.340sec	1.557sec	2.963sec	34.966sec	1min 15.948sec
			total	**0.493sec**	**1.888sec**	**3.393sec**	**36.604sec**	**1min 18.331sec**
		7	*genhists file*	0.329sec	0.721sec	0.943sec	3.619sec	5.312sec
			gentauk file	0.773sec	3.550sec	6.601sec	1min 27.246sec	2min 59.515sec
			total	**1.102sec**	**4.271sec**	**7.544sec**	**1min 30.865sec**	**3min 4.827sec**
		8	*genhists file*	1.096sec	2.316sec	3.142sec	11.702sec	19.384sec
			gentauk file	2.563sec	11.615sec	20.660sec	4min 45.257sec	10min 48.107sec
			total	**3.659sec**	**13.931sec**	**23.802sec**	**4min 56.959sec**	**11min 7.491sec**
		**Total running time**		**7.689sec**	**26.930sec**	**45.883sec**	**8min 56.710sec**	**19min 18.815sec**
our approach	LINUX	8	*1_fa.py file*	**51sec**	**1min 12sec**	**1min 6sec**	**3min 46sec**	**5min 55sec**
	WINDOWS	8	*1_fa.py file*	**1min 10sec**	**1min 45sec**	**1min 30sec**	**5min 53sec**	**11min 6sec**

REFERENCES

Heady, R. B. and J. L. Lucas (1997). "PERMAP: An interactive program for making percentual maps." Behavior Research Methods, Instruments & Computers 29 (3): 450-455.

Soares, I., A. Amorim and A. Goios (2011). "A new algorithm for mtDNA sequence clustering." Forensic Science International: Genetics Supplement Series **3** (1): e315-e316.

Stone, A. C., F. U. Battistuzzi, L. S. Kubatko, G. H. Perry, Jr., E. Trudeau, H. Lin and S. Kumar (2010). "More reliable estimates of divergence times in Pan using complete mtDNA sequences and accounting for population structure." Philosophical transactions of the Royal Society of London. Series B, Biological sciences **365** (1556): 3277-3288.

Tamura, K., J. Dudley, M. Nei and S. Kumar (2007). "MEGA4: Molecular Evolutionary Genetics Analysis (MEGA) software version 4.0." *Molecular Biology and Evolution* **24** (8): 1596-1599.

van Oven, M. and M. Kayser (2009). "Updated comprehensive phylogenetic tree of global human mitochondrial DNA variation." Human Mutation **30** (2): E386-E394.

Zsurka, G., T. Kudina, V. Peeva, K. Hallmann, C. E. Elger, K. Khrapko and W. S. Kunz (2010). "Distinct patterns of mitochondrial genome diversity in bonobos (Pan paniscus) and humans." BMC evolutionary biology **10**: 270.
